# ABA-Dependent Regulation of Calcium-Dependent Protein Kinase Gene *GmCDPK5* in Cultivated and Wild Soybeans

**DOI:** 10.3390/life12101576

**Published:** 2022-10-11

**Authors:** Galina N. Veremeichik, Evgenia V. Brodovskaya, Valeria P. Grigorchuk, Ekaterina S. Butovets, Ludmila M. Lukyanchuk, Victor P. Bulgakov

**Affiliations:** 1 Federal Scientific Center of the East Asia Terrestrial Biodiversity, Far Eastern Branch of the Russian Academy of Sciences, 159 Stoletija Str., 690022 Vladivostok, Russia; 2 Federal Scientific Center of Agrobiotechnology of the Far East Named after A.K. Chaika, 692539 Ussuriysk, Russia

**Keywords:** *Glycine* *max*, wild soybean, calcium-dependent protein kinase, abiotic stress, abscisic acid

## Abstract

Calcium-dependent protein kinases (CDPKs) regulate plant development and stress responses. However, the interaction of these protein kinases with the abscisic acid (ABA) stress hormone signalling system has not been studied in detail. In *Arabidopsis*, AtCPK1 plays an important role in the acclimation of plants to environmental stresses. Phylogenetic and molecular analyses showed that, among 50 isoforms of *Glycine max* (L.) Merrill CDPKs, the GmCDPK27/GmCDPK48, GmCDPK5/GmCDPK24, and GmCDPK10/GmCDPK46 paralogous pairs were the isoforms most related to AtCDPK1. We investigated the expression of the corresponding six *GmCDPKs* genes during treatment with cold, heat, and salt stress. Wild soybean was the most resistant to stresses, and among the three cultivars studied (Sfera, Hodgson, and Hefeng25), Sfera was close to the wild type in terms of resistance. *GmCDPK5* and *GmCDPK10* were the most responsive to stress treatments, especially in wild soybean, compared with cultivars. Among the studied *GmCDPK* isoforms, only *GmCDPK5* expression increased after treatment with abscisic acid (ABA) in a dose- and time-dependent manner. Targeted LC-MS/MS analysis of endogenous ABA levels showed that wild soybean and Sfera had nearly twice the ABA content of Hodgson and Hefeng25. An analysis of the expression of marker genes involved in ABA biosynthesis showed that *GmNCED1*-gene-encoding 9-cis-epoxycarotenoid dioxygenase 1 is induced to the greatest extent in wild soybean and Sfera under salt, cold, and heat exposure. Our data established a correlation between the induction of *GmCDPK5* and ABA biosynthesis genes. *GmCDPK5* is an interesting target for genetic and bioengineering purposes and can be used for genetic editing, overexpression, or as a marker gene in soybean varieties growing under unfavourable conditions.

## 1. Introduction

Adverse climate conditions, such as cold, heat, sudden temperature fluctuations, and salinity, limit sustainable crop production. The rapid development of genome editing technology and application of the marker-assisted selection, as a promising solution to this problem, requires further study in soybean [1]. Soybean (*Glycine max* (L.) Merrill; family Leguminosae) is one of the most important crop plants [2] and a model system due to the availability of whole-genome databases [3]. The search for markers or target genes is relevant in connection with the existing problems of the soybean “green revolution” [4]. One of the main problems is genome duplication and the abundance of gene paralogs, which makes it difficult to select an exact target [5].

Calcium-dependent protein kinases (CDPKs) can be used as markers or target genes to improve crop stress tolerance, as they regulate plant growth and development, as well as response to abiotic stresses. The role of CDPK in stress resistance is realized through phosphorylation of numerous target proteins [6,7]. CDPKs are involved in drought and salt stress signalling by activating anionic and potassium currents via the S-type ion channels SLAC1 and SLAH3, the outward K^+^ channel GORK, and the inward K^+^ channel KAT1/2 [7]. CDPKs have also been shown to phosphorylate C2H2 zinc finger transcription factors such as AtDi19, constituting CDPK-Di19 complexes involved in drought response [7]. CDPKs regulate abscisic acid (ABA) signalling by phosphorylation of ABA-responsive element binding factors (ABFs) as well as ABA signalling components located upstream of ABFs (mainly protein phosphatases 2C, PP2Cs). Other direct targets of CDPK are phenylalanine ammonia-lyase (PAL), which is involved in flavonoid biosynthesis, and respiratory burst oxidase homologue protein D (RBOHD). RBOHD is involved in reactive oxygen species (ROS) signalling and ROS wave propagation, thus creating the necessary conditions for acclimation [6,7].

*AtCPK1* (the name of the corresponding protein is AtCDPK1; http://www.uniprot.org/uniprot/Q06850, accessed on 3 August 2022) is one of the most studied *CDPK* genes in plants. The *AtCPK1* gene is a positive regulator of the response to biotic stress [8], pathogen resistance via ROS production [7], salt, temperature, and drought tolerance [9,10,11], and phytoalexin biosynthesis, including isoflavonoids of *G*. *max* [12,13,14,15,16]. In this respect, *GmCDPK*s, which are related to *AtCPK1*, are the most attractive targets for cultivar improvement.

Among 50 isoforms of the *G. max CDPK* genes, the forms most related to *AtCPK1* are three pairs of paralogs: *GmCDPK5*/*GmCDPK24*, *GmCDPK10/GmCDPK46*, and *GmCDPK27/GmCDPK48* (isoform numbering is presented according to the Hettenhausen et al. [17] classification). *GmCDPK27* is expressed in the roots, stems, and leaves of mature soybean plants, whereas *GmCDPK5* is expressed only at the cotyledon stage [17]. The expression level of *GmCDPK27* increased no more than twofold under biotic and drought stress treatments [17]. Salicylic acid and biotic treatments induced *GmCDPK5* expression no more than twofold. Among other *GmCDPK* genes, *GmCDPK5* is the most inducible under ABA and drought stress (more than 30- and 10-fold, respectively; [17]). The expression of the *GmCDPK24* gene, a paralog of *GmCDPK5*, also increased under the same stress conditions but to a much lesser extent (no more than twofold; [17]). The abundance of *GmCDPK* isoforms, closely related to *AtCPK1*, significantly complicates the selection of the most useful targets; it is unclear which of these six isoforms is involved in defence responses and enhances stress tolerance. In the present study, the expression of these three pairs of paralogs was investigated under severe and moderate temperature and salt stress.

ABA is an essential plant hormone that triggers the responses of plants to adverse climate conditions by regulating the expression of genes involved in the process of acclimation [18]. The first step in ABA biosynthesis from zeaxanthin is catalysed by zeaxanthinepoxidase (ZE, EC 1.14.13.90; [19]). Key enzymes involved in stress-induced ABA biosynthesis are 9-*cis*-epoxycarotenoid dioxygenases (NCED, EC 1.13.11.51), namely isoforms closely related to *AtNCED3* that regulate endogenous ABA levels and promote transcription of ABA-inducible genes [20,21].

Thus, the present study focused on a complex analysis of the relationships between GmCDPKs related to AtCPK1, ABA content and biosynthesis during abiotic stress treatments. Wild soybean (*Glycine soja* Sieb. and Zucc., family Leguminosae) from the northernmost area and cultivated soybeans of zoned varieties from American (‘Hodgson’) and Chinese (‘Hefeng25′) selections were used in a comparative analysis. In addition, the Russian variety (‘Sfera’) was chosen due to the climatic conditions in the risk zone (sudden changes in temperature and humidity with a predominance of low temperature and high humidity) in which both wild soybeans and this variety grow in the Russian Far East (43°48′ north latitude, 131°58′ east longitude). In our previous work, the Sfera variety was shown to be more productive in these growing conditions compared with the zoned varieties [22]. In the present study, the levels of abiotic stress tolerance of cultivated and wild soybeans and the role of CDPKs and ABA in these acclimation processes were investigated. In addition, we conducted a comparative analysis of the expression of the nearest homologues of *AtCPK1* among six *GmCDPKs* paralogs to determine the most promising target for genetic improvement of soybean varieties growing under unfavourable conditions

## 2. Materials and Methods

### 2.1. Plants Material, Cultivation, and Experimental Design

Soybean plants and seeds were used, as previously described [22]. Wild soybean (*Glycine soja* Sieb. and Zucc.) seeds were collected from wild growing (south of the Primorskii region, Ussuriysk district, Russia) plants and determined in the Federal Scientific Centre of Agrobiotechnology. Varieties of soybean (*Glycine max* (L.) Merrill) plants of American (*cv*. Hodgson), Chinese (*cv*. Hefeng25) and domestic (*cv*. Sfera) selection were taken from the seed collection of the Federal Scientific Centre of Agrobiotechnology in the Far East named after A.K. Chaika, Ussuriysk.

Soybeans seeds were germinated and cultivated in vitro for elimination of contamination in culture vessels on MS/2 medium containing 10 g·L^−1^ sucrose [23] in climate chamber (KS-200, Russia) for 45 days. Conditions of cultivation were as follows: photoperiod, 16/8 h with illumination in the daytime 140 µmol m^−2^ s^−1^; temperature, 24/22 °C; humidity, 70%. For long-term analysis (90 days), plants were grown in the experimental field during three seasons as previously described [22].

To analyse the tolerance of the studied plants to prolonged temperature stress, the plants were germinated and grown in vitro under the same conditions, excluding the temperature regime during day/night: control, 24/22 °C; cold, 16/12 °C; heat, 36/34 °C. The effect of salinity stress on long-term growth and biomass accumulation of wild and cultivated soybean plants was investigated by adding 30, 60, 90, and 120 mM NaCl (Panreac, Barcelona, Spain) during the 35-day period of cultivation. Samples were harvested from 35-day-old plantlets, weighed, and total RNA samples were extracted.

To study the germination of wild and cultivated soybeans seeds, sterile plates were used with sterile paper disks soaked in distilled water. The conditions of germinations were as follows: photoperiod, 16/8 h; illumination in the daytime, 140 µmol m^−2^ s^−1^; humidity, 70%. Temperatures for control conditions, cold and heat treatments were 23 °C, 6 °C, 37 °C, and 39 °C, respectively, for 72 h. For analysis of seed germination under salinity, paper disks were soaked in distilled water with 0, 240, and 360 mM NaCl. Sprouts longer than 10 mm were counted and photographed on the 3rd day in all analysed seedlings growing both in control and under stress conditions. For temperature and salt stress treatments, experiments were repeated three times with ten technical replicates.

To analyse the patterns of *GmCDPKs* expression under short and strong stress exposure, the following experiments were carried out. The 35-day-old plants growing in vitro were transferred in the liquid medium for 24 h for adaptation; then, plants were treated with 0 and 150 mM NaCl for 1 and 4 h. For severe cold and heat treatments, plants were placed for 1 and 4 h at 6 °C and 40 °C, respectively. Then, plants samples were used for total RNA extraction. These experiments were repeated three times with three technical replicates [11].

The effect of ABA treatment on *CDPK* expression was tested on 35-day-old soybean plants. Plants were sprayed with freshly prepared 1- and 5-mM ABA (Sigma, Saint Louis, MO, USA) solutions in 10% ethanol; mRNA levels of *GmCDPKs* were measured by real-time PCR after 1, 4, and 24 h. Plants sprayed with 10% ethanol and untreated plants were used as controls. *GmCDPK5* expression (indicated as normalized relative fold expression) in wild and cultivated soybean plants such as varieties of American (Hodgson), Chinese (Hefeng25), and Russian (Sfera) selection was measured after treatment of 1 mM of ABA for 1 and 4 h. Plants were grown in vitro for 35 days in control condition (24/22 °C). All kinds of ABA treatments were repeated three times in three independent experiments (biological replicates) and qPCR of each biological replicates were performed in three technical replicates.

### 2.2. Molecular and Phylogenetic Analysis of the GmCDPKs and the Design of Unique Primer Pairs

In this study, the closest homologues of AtCPK1 (GenBank accession number, AT5G04870; http://www.uniprot.org/uniprot/Q06850, accessed on 3 August 2022) in *G*. *max* were *GmCDPK5*, *GmCDPK10*, and *GmCDPK27*, and their paralogs, *GmCDPK24*, *GmCDPK46*, and *GmCDPK48*, respectively, according to a previous study [17], and corresponding gene numbering was used. Sequences of the *G. max* CDPK genes were retrieved from GenBank and Phytozome databases; corresponding accession numbers are listed in Supplementary Table S1.

For alignment of nucleic acid and amino acid sequences, Clustal W and ClustalX programs were used. Based on nucleic acid alignment, there were some inconsistencies between sequences from GenBank and Phytozome databases in the 5′-UTR of some CDPKs. Therefore, to design primer pairs for qPCR, only the reliable CDS portion of *CDPK* genes was used. To distinguish the expression of paralog pairs (*GmCDPK5*/*GmCDPK24*, *GmCDPK10*/*GmCDPK46,* and *GmCDPK27*/*GmCDPK48*), primer pairs were created at the sites of maximum differences according to nucleic acid alignment (Supplementary Figure S1).

Using Clustal Omega for multiple sequence alignment (MSA; https://www.ebi.ac.uk/, accessed on 28 March 2017), pairwise analysis was performed to create a percent identity matrix. All functional domains of CDPKs were analysed using Prosite Expasy. Phylogenetic trees based on full-length and partial protein sequences, corresponding to CDPK domains, were generated using MABL tools (http://www.phylogeny.fr/advanced.cgi, accessed on 7 September 2022 [24]) with the following parameters: multiple alignment—MUSCLE; alignment curation—Gblocks; construction of phylogenetic tree—PhyML; statistical tests for branch support—bootstrapping procedure; number of bootstraps—100; substitution model—Dayhoff.

### 2.3. RNA Extraction, cDNA Synthesis, and qPCR Reaction

LiCl method with DNAse processing stage (15 min on 37 °C with 1 uL of RNAse free DNAse, Panreac, Spain) was used for isolation of total RNA from *G. max*. Integrity and purity of RNA was analysed using Microcapillary electrophoresis chips (Experion, Bio-Rad Laboratories, Hercules, CA, USA). The first strand of complementary DNA (cDNA) from RNA (2.5 µg) was obtained using Oligo-d(T)_15_ primer (0.5 ng) and RT reagent Biolabmix (Novosibirsk, Russia) following the manufacturer’s protocol. In addition, we used each RNA sample reacted in the absence of M-MLV enzyme as a negative (RNA-RT) control. All procedures have been described in detail [22].

Quantitative real-time PCR (qPCR) analysis was performed using a CFX96 (Bio-Rad Laboratories, Inc., Hercules, CA, USA) with 5 x SYBR green PCR master mix Biolabmix (Novosibirsk, Russia) according to the manufacturer’s protocol. As a reference gene for soybean, we used the gene-specific primer pairs to eukaryotic elongation factor 1-beta (*GmELF1*) and cyclophilin protein folding (*GmCYP*) as described previously [16]. Specific primers pairs for qPCR analysis of *GmCDPKs* gene expression and expression of genes encoding enzymes involved in ABA biosynthesis are listed in Supplementary Tables S1 and S2. Design of primer pairs and analysis of secondary structures was performed using ClustalX2 and Gene Runner programs. Data analysis was performed using CFX Manager Software (Version 1.5; Bio-Rad Laboratories Inc., Hercules, CA, USA). The relative expression was determined by normalization to the reference gene and calculated by the 2^−ΔΔCt^ method [25]. Three biological replicates derived from three separate RNA extractions, with three technical replicates analysed. Controls without a template were included to confirm the absence of contamination. The absence of primer–dimer artefacts or non-specific products was confirmed by a melting curve analysis culminated each run. Other essential [26] parameters of the qPCR are listed in Supplementary Tables S1 and S2**.** Heat maps were built with http://www.heatmapper.ca/tools, accessed on 7 September 2022 [27].

### 2.4. ABA Measurement

#### 2.4.1. Chemicals

All solvents were of high-performance liquid chromatography (HPLC) grade. An analytical standard of ABA was obtained from Sigma-Aldrich (Saint Louis, MO, USA). The standard was dissolved in methanol for a stock concentration of 2.0 mg/mL and stored at 4 °C. Working standard solutions of lower concentrations were prepared by diluting the stock solution with methanol prior to use.

#### 2.4.2. Sample Preparation for HPLC-MS/MS

Plant material (1 g, accurate weight) was harvested, ground in liquid nitrogen to produce a fine powder, quantitatively divided into two parts, and immediately covered with 1 mL extraction solvent (for the first part—isopropanol: methanol: glacial acetic acid, 80:20:1 (*v*/*v*/*v*); for the second part—the same solvent with the addition of a standard solution of ABA at a final concentration of 1.0 ng·mL^−1^). Samples were mixed with a vortex, sonicated at room temperature for 5 min, incubated on ice for 20 min, and centrifuged (15,000× *g*, 10 min, 4 °C). The supernatant was collected, cleared with a 0.45 μm membrane (Millipore, Bedford, MA, USA), and immediately used for HPLC-MS/MS analysis [28].

#### 2.4.3. HPLC-MS/MS Analysis

HPLC–MS/MS analysis of all samples was performed at the Instrumental Centre of Biotechnology and Gene Engineering of Federal Scientific Center of the East Asia Terrestrial Biodiversity. A measure of 5 μL of each extract was injected into a 1260 Infinity analytical HPLC system (Agilent Technologies, Santa Clara, CA, USA). An analytical Zorbax C18 column (150 mm, 2.1 mm i.d., 3.5 μm part size, Agilent Technologies, Santa Clara, CA, USA) was used for separation. The column was maintained at 40 °C and eluted at a flow rate of 0.20 mL·min^−1^. The mobile phase consisted of water (solvent A) and acetonitrile (solvent B), each containing 0.1% (*v/v*) formic acid. A linear gradient of solvent B was as follows: 0 min, 40% B; 0–5 min, 40–55% B; 5–6 min, 55–100% B; 6–9 min, 100% B. The column elution was analysed by tandem mass-spectrometry using an ion trap (IT) instrument Bruker HCT ultra PTM Discovery System (Bruker Daltonik GmbH, Bremen, Germany) equipped with an electrospray ionisation (ESI) source and operated in the negative ion polarity. The following ESI settings were used: capillary voltage, 4 kV; end plate offset voltage, −500 V; capillary exit voltage, −110 V; nebuliser pressure, 25 psi; drying gas flow rate, 8 L·min^−1^; and temperature, 325 °C. Nitrogen gas was used as a nebuliser and drying gas. The quantitative data were obtained in manual MS^2^ mode with the isolation of precursor mass at *m/z* 263 (for ABA [M-H]^−^) and isolation width at *m/z* 2.0. The fragmentation amplitude was set to 1.5 V. Bruker Daltonics Compass 1.3 esquire control software (v.6.2.581.3 and v.4.0.234.0, accessed on 2008) was used for instrument control, data acquisition, and data processing.

### 2.5. Statistical Analysis

All values are expressed as mean ± SE. For statistical evaluation of significance, Student’s *t*-test was used to compare the two independent groups. Analysis of variance (ANOVA) was used for comparison among multiple data. For the inter-group comparison, Fisher’s protected least significant difference (PLSD) post hoc test was employed. The level of statistical significance was set at *p* < 0.05.

## 3. Results

### 3.1. Comparative In Vitro Analysis of Abiotic Stress Resistance of Wild and Cultivated Soybean Plants

**Figure 1:**
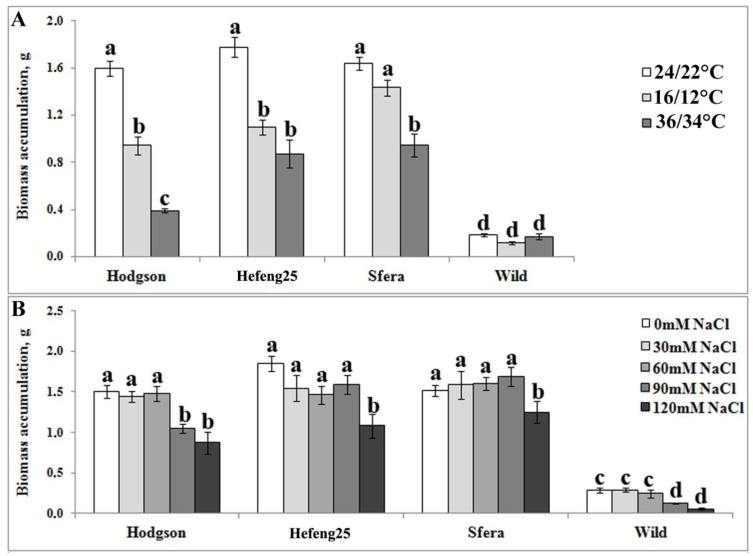
Biomass accumulation of wild and varietal soybean plants (Hodgson, Hefeng25, and Sfera) growing under cold or warm temperature conditions (**A**) or salt treatment (**B**). Plants were growth in vitro under controlled condition for 35 days. Control conditions, cold and heat treatment temperatures (day/night) were 24/22 °C, 16/12 °C, and 36/34 °C, respectively. For salt treatment, 0, 30, 60, 90, and 120 mM NaCl were added to the culture medium. Experiments were repeated three times in ten replicates for each variant. The data are presented as mean ± SE. Different letters above the bars indicate significantly different means (*p* < 0.05), Fisher’s LSD.

Before analysis of GmCDPK expression after stress treatment, the level of tolerance of wild and cultivated soybeans to abiotic stress treatment was tested. The tolerance of the most northern wild soybean, zoned cultivars (Hodgson and Hefeng25), and the cultivar Sfera, which were selected for the present climate conditions, was compared. Long-term (35 days) cold (16/12 °C) and heat (36/34 °C) treatments did not affect the growth and biomass accumulation of the wild soybean (Figure 1A). However, cold affected the growth of zoned cultivars Hodgson and Hefeng25 but not Sfera. Heat stress decreased the growth of all cultivars; however, the greatest decrease was observed for Hodgson (Figure 1A). Under salt treatment, growth of wild and cultivated soybeans was affected when the NaCl concentration reached 90 mM (for Hodgson and wild soybean) and 120 mM for Hefeng25 and Sfera (Figure 1B).

**Figure 2:**
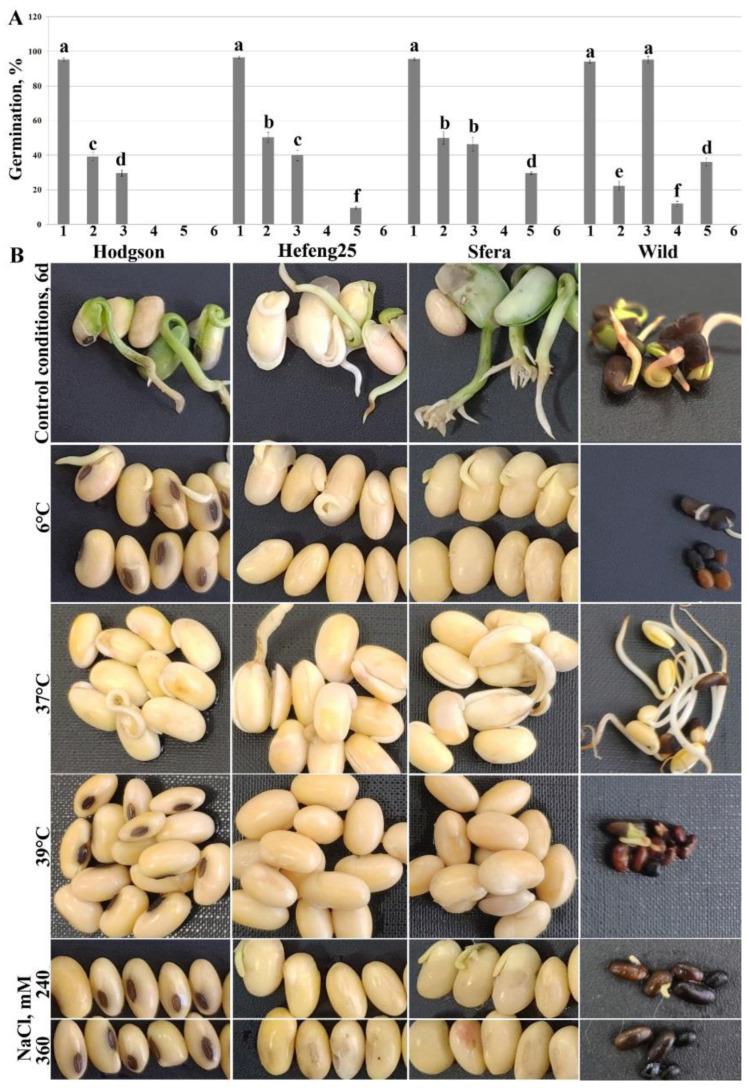
Characterization of germination (**A**) under severe temperature and salinity treatment of the wild and cultivated soybean plants: Hodgson, Hefeng25, and Sfera. Seeds were germinated in wet plates under controlled condition for 3 days. Control (1), cold (2), and heat (3 and 4) treatment temperatures were 23 °C, 6 °C, 37 °C, and 39 °C, respectively, for 72 h. For salinity treatment (5 and 6) seeds were germinated at the plates containing 240 and 360 mM NaCl, respectively, at 23 °C for 72 h. After 72 h, results were photographed (**B**). Experiments were repeated three times in ten replicates for each variant. The data are presented as mean ± SE. Different letters above the bars indicate significantly different means (*p* < 0.05), Fisher’s LSD.

Severe temperature and salt effects were investigated using germination assay. Seeds of wild and cultivated soybeans were germinated under extremely low (6 °C) and high (37 and 39 °C) temperatures and high salinity (240 and 360 mM NaCl). Cold decreased the germination of all studied varieties more than twofold; high temperature (37 °C) affected soybean cultivar germination but did not influence the wild soybean. Increasing the temperature up to 39 °C completely inhibited soybean cultivar germination and reduced wild soybean germination fivefold (Figure 2A,B). High salinity (240 mM) affected the germination of Hefeng25, Sfera, and wild soybean more than twofold and completely inhibited Hodgson. An increase in the NaCl concentration to 360 mM completely suppressed the germination of all cultivars studied and wild soybean (Figure 2A,B). After three days of severe salt stress treatment, the non-germinated wild soybean seeds were still viable, but the cultivars died.

### 3.2. Molecular and Phylogenetic Analysis of G. max AtCDPK1 Homologues

An important goal was to identify the most promising *GmCDPK* isoforms for genetic manipulation based on the well-known properties of *AtCPK1* (synonym: *Ak1*, the name of the corresponding protein is AtCDPK1; https://www.uniprot.org/uniprotkb/Q06850/entry, accessed on 3 August 2022). Previously, *GmCDPK5* expression was shown to be the most induced by ABA [17]. On the other hand, *GmCDPK5* is not the most related *AtCPK1* isoform due to the polyploidy of the *G. max* genome. We focused on studying the expression patterns of three pairs of *GmCDPKs* paralogs closest to *AtCPK1* among 50 isoforms of *GmCDPKs*—*GmCDPK5*, *GmCDPK10*, and *GmCDPK27*—and their paralogs *GmCDPK24*, *GmCDPK46*, and *GmCDPK48*, respectively, according to previous studies [17]. Phylogenetic analysis of these six *G. max* CDPK isoforms and 32 *A*. *thaliana* CDPKs showed that all six GmCDPKs clustered together with AtCDPK1 and AtCDPK2 (Supplementary Figure S2). Percent Pairwise Identity Matrix of GmCDPK sequences based on alignment of multiple nucleic acid sequences (parts of CDS) and amino acids was calculated using Clustal Omega. The analysis showed that GmCDPK48 (73.98 and 81.5%, paralog of GmCDPK27) was the most similar to AtCDPK1 (Supplementary Table S3, highlighted in green). GmCDPK46 (65.98 and 64.04%, paralogs of GmCDPK10) was the least similar (Supplementary Table S3, highlighted in red). The GmCDPK46/GmCDPK48 and GmCDPK27/GmCDPK46 pairs were the most distant from each other based on nucleic acid and amino acid alignment (67.65% and 65.74%), respectively (Supplementary Table S3**,** highlighted in blue).

Before performing a detailed analysis based on the full-length and domain parts, the domain structures of each form were analysed using the Expasy Prosite tools. The analysis showed that all six isoforms had a complete set of the domains typical for CDPK: N-terminal variable domain, protein kinase domain, junction, and CaM-like domain with EF hands (Supplementary Figure S3). However, the paralogs GmCDPK10/GmCDPK46 had an incomplete first EF-hand in the CaM-like domain with the substitution of the essential EF-hand amino acid E (underlined) in the EF-hand sequence DTDNSGQITLEK, which did not satisfy the following condition: D-x-[DNS]-{ILVFYW}-[DENSTG]-[DNQGHRK]-{GP}-[LIVMC]-[DENQSTAGC]-x(2)-[DE] (Supplementary Figure S3).

A more detailed phylogenetic analysis of full-length GmCDPKs and AtCDPK1 showed that among all six isoforms, the pair GmCDPK27/GmCDPK48 was the most related to AtCDPK1, and the pair GmCDPK10/GmCDPK46 was the least related (Supplementary Figure S4A). Phylogenetic analysis of CDPKs based on partial amino acid sequences corresponding to domains showed similar results (Supplementary Figure S4B–D), except for the tree based on the junction sequence. In this case, the pair GmCDPK5/GmCDPK24 was more related to AtCDPK1 than the pair GmCDPK27/GmCDPK48 (Supplementary Figure S4E).

### 3.3. CDPK Expression during Growth Phases

Differential expression of six *GmCDPKs* was analysed during the growth phases. Previous RNA sequencing data from expression profiles showed a high level of expression of the GmCDPK27/GmCDPK48 paralog pair and the absence of expression of the other two pairs, GmCDPK5/GmCDPK24 and GmCDPK10/GmCDPK46, in different tissues and at different stages of growth [17]. We performed a quantitative PCR analysis starting from 5-day-old seedlings in the cotyledon growth phase (5–25 days) to maturation (90 days) of three soybeans varieties and wild soybeans grown in vitro (0–45 days) and in the field (90 days).

**Figure 3:**
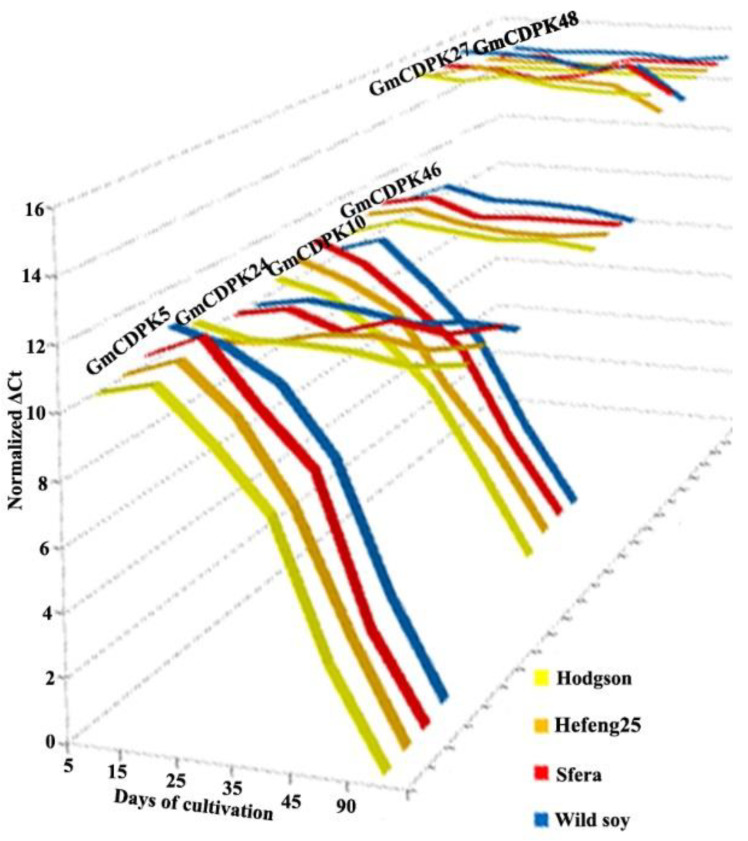
Expression dynamics of CDPK genes in *G. max*. mRNA levels were measured by real-time PCR in wild and cultivar soybean varieties (Hodgson, Hefeng25, and Sfera) at different growth fazes, from 5 to 90 days. Data are presented as the mean of normalized ΔCt; each point for each variant was measured in three independent experiments (biological replicates) and three technical replicates. The statistical significance of the data presented is shown in the Supplementary Table S4 (*p* < 0.05), Fisher’s LSD.

The expression level of the most *AtCPK1*-related paralog pair, *GmCDPK27/GmCDPK48*, was high and similar to each other (Figure 3). The mean Ct value of these two paralogs was about 27 cycles when the amount of the analysed cDNA samples corresponded to 24–26 Ct of housekeeping genes (Supplementary Table S4). At the same time, the expression of the two other paralog pairs was completely different. The *GmCDPK5* and *GmCDPK10* isoforms were slightly expressed from 5 to 35 days with a significant tendency to decrease expression after 45 days of growth: from 30 to 42 cycles for *GmCDPK5* and from 33 to 38 cycles for *GmCDPK10* (Figure 3, Supplementary Table S4). Their paralogs, *GmCDPK24* and *GmCDPK46*, had an equally slight expression level during growth, close to 31 and 30 cycles, respectively (Figure 3, Supplementary Table S4). The comparison of such different expression levels among the six studied *GmCDPK* isoforms by classical methods (2^−^^ΔΔCt^) was difficult to assess due to the large fold change in expression (more than 2^15^). Therefore, the data are presented as the normalised ΔCt in Figure 3 and additionally as the normalised mean Ct, with ANOVA analysis of statistical validity (Supplementary Table S4).

### 3.4. CDPK Expression under Short- and Long-Term Abiotic Stress Treatment

**Figure 4:**
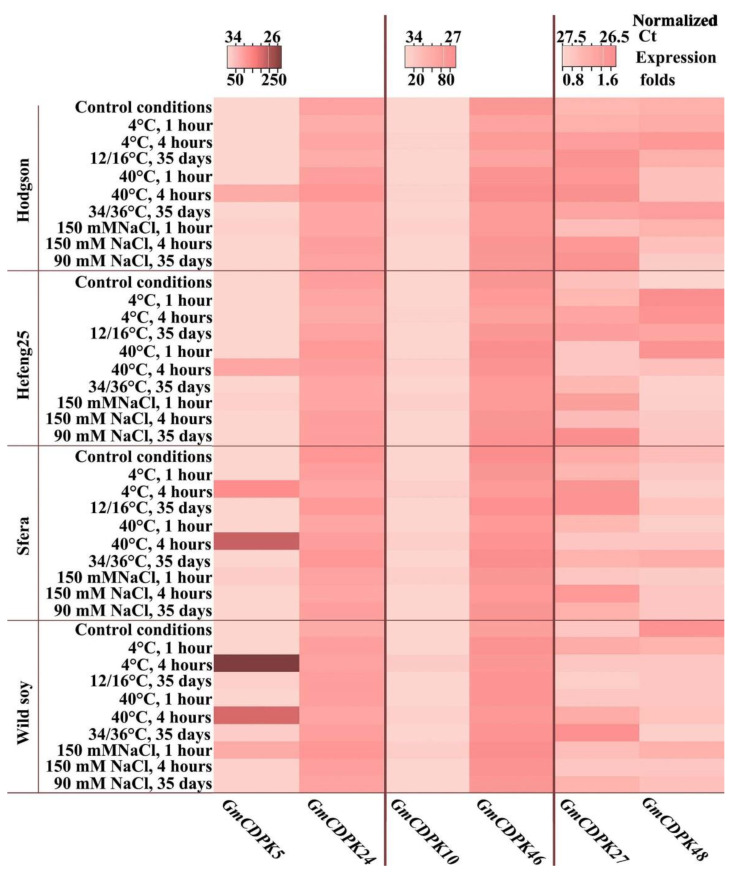
*GmCDPK* gene expression under abiotic stress treatment. mRNA levels were measured by real-time PCR (normalized relative fold expression) in wild and cultivar soybean varieties (Hodgson, Hefeng25, and Sfera). Plants were grown in vitro for 35 days in control condition (22/24 °C), in condition of long middle stress treatment: cold (12/16 °C), heat (34/36 °C), and salinity (90 mM NaCl). In addition, 35-day-old plants of each variant were treated with short severe stresses: cold (4 °C), heat (40 °C), and salt treatment (150 mM NaCl) for 1 and 4 h. All stress treatments were repeated three times in three independent experiments (biological replicates) and qPCR measurements of each biological replicate were performed in three technical replicates. Data are presented as a heatmap calculated as 2^−^^ΔΔCt^ from qPCR data. Different colours indicate significantly different means (*p* < 0.05), Fisher’s LSD.

The expression patterns of three pairs of *GmCDPK* gene paralogs were studied using the same controls and stress-treated samples as described in Section 3.2. The analysis showed that the expression of the GmCDPK27/GmCDPK48 paralog pair did not change significantly or more than twofold under stress conditions, compared with control conditions (Figure 4, Supplementary Table S5).

The expression of *GmCDPK10* was sensitive only to 4 h temperature stress and 1 h salinity stress. The change in expression was ten-fold compared with control conditions for wild and cultivated soybeans (Figure 4, Supplementary Table S5). Under stress, the expression of its paralog, *GmCDPK46*, was not changed. Similarly, stress treatments affected the expression of the paralog pair *GmCDPK5*/*GmCDPK24*. Stress treatment did not affect *GmCDPK24* expression in any of the samples, while *GmCDPK5* expression patterns under stress differed greatly. In Hodgson and Hefeng25, the expression of *GmCDPK5* increased from 3- to 6-fold during short-term cold treatment (from 1 to 4 h). After 1 h of heating, the expression increased almost 6-fold, and after 4 h, it increased almost 100-fold. Salinity treatment led to a 10-fold increase in *GmCDPK5* expression after 1 h of treatment with a subsequent decrease to the baseline; long-term stress treatment did not significantly change *GmCDPK5* expression (Figure 4, Supplementary Table S5).

In wild soybean and Sfera, *GmCDPK5* expression dramatically increased (more than 100-fold) under both short-term temperature stressors (4 h after treatment). Salinity resulted in a strong increase in *GmCDPK5* expression by more than 20 and 70 times after 1 h of salt treatment in wild and Sfera soybeans, respectively (Figure 4, Supplementary Table S5). The long-term stress treatment slightly increased *GmCDPK5* expression in both Sfera and wild soybean.

### 3.5. ABA Levels and 9-Cis-Epoxycarotenoid Dioxygenases Gene Expression

To determine the role of ABA in the resistance of soybean varieties to abiotic stress treatment, we analysed the baseline levels of endogenous ABA and the expression of genes encoding key components of the ABA biosynthetic pathway.

#### 3.5.1. ABA Determination

**Figure 5:**
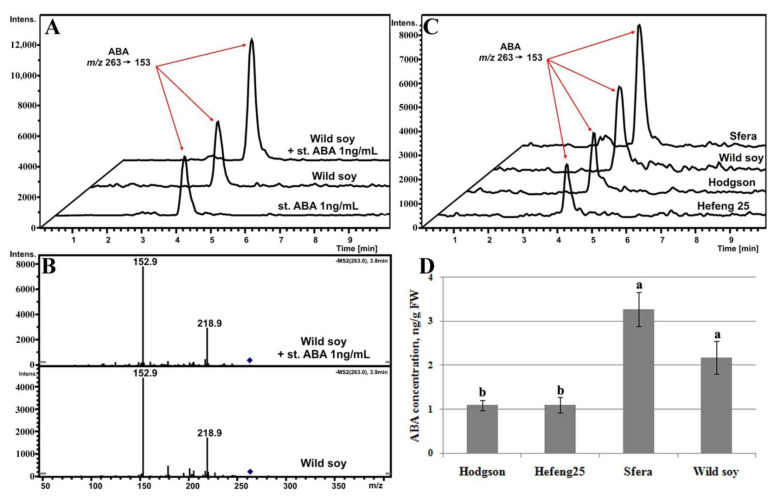
Targeted LC–MS/MS analysis of the crude extracts of wild and cultivated soybean plants recorded in MS^2^ mode of precursor ions at *m/z* 263. Chromatograms of recovered ions of *m/z* 263→153 are shown (**A**,**C**). MS/MS product-ion mass spectra of the precursor ion with *m/z* 263 (**B**). The resulting ABA concentrations in analysed samples (**D**) are presented in ng·g^−1^ FW. Plants of wild and cultivated soybean plants were grown in vitro for 35 days in control condition (23 °C). Data obtained in three independent experiments with three technical replicates for each plant are presented as mean ± SE. Different letters above the bars indicate statistically significant differences of means (*p* < 0.05), Fisher’s LSD.

The LC–MS/MS method was used for profiling and quantification of ultra-trace ABA content in crude extracts of 35-day-old wild and cultivated soybean grown in vitro. The chromatographic conditions were optimised with 100 ng·mL^−1^ standard solution to minimise the analysis time. The analysis was carried out for 10 min, the ABA retention time was 3.6–4.2 min. The fragmentation pattern of precursor ions at *m/z* 263 for ABA [M-H]^−^ was studied. The two main diagnostic fragments at *m/z* 219 and 153 were observed in the MS/MS spectrum of ABA (Figure 5B) with good intensity and low background for standard solutions (from 0.5 to 10 ng·mL^−1^) and sample extracts. To increase the sensitivity for ABA determination, the tandem mass spectrometry technique in manual MS^2^ mode was chosen, in which only the ions of interest were tracked. The characteristic ions at *m/z* 153 were selected to generate chromatograms of the extracted ions (Figure 5A–C) and used for quantification. The standard addition method was used (Figure 5A), where the ABA standard solution of a certain concentration was added directly to the analysed sample to minimise matrix effects. Two analyses in two parallel experiments were carried out for each sample, the first with the additive and the second without the additive. ABA quantification was performed directly using a straight-line calibration curve (from 0.5 to 10 ng mL^−1^). The standard solution was analysed at the beginning and end of the experiment to verify the stability of calibration dependence.

The analysis showed that the initial level of endogenous ABA under control conditions was more than twice as high in wild soybean and Sfera variety compared with Hodgson and Hefeng25 varieties (Figure 5D).

#### 3.5.2. ABA-Biosynthesis-Related Gene Expression

**Figure 6:**
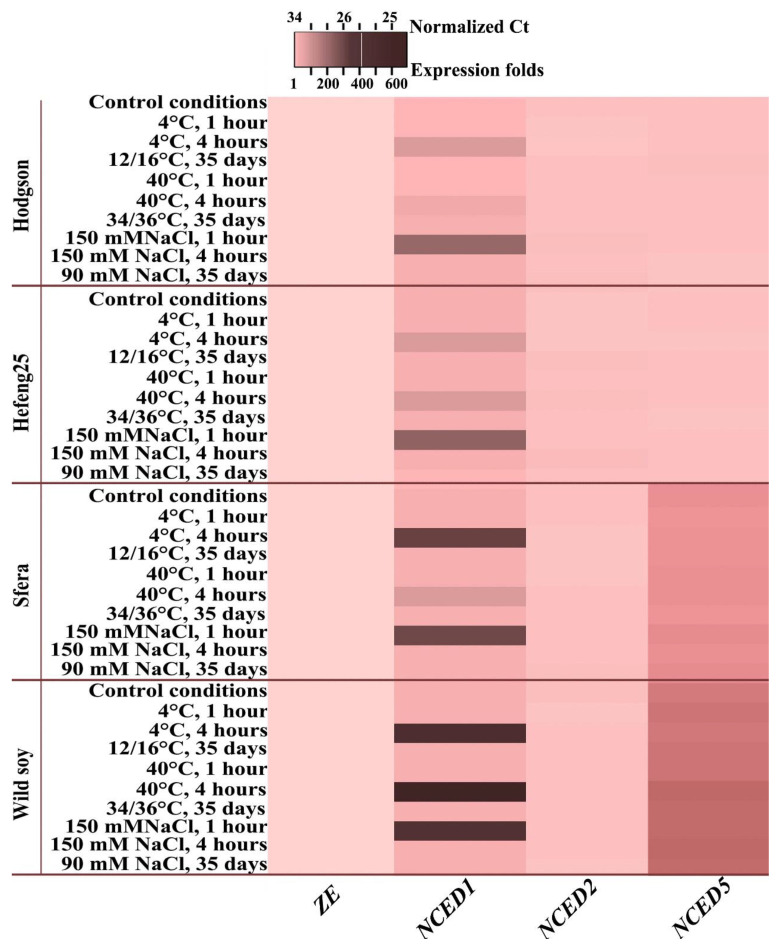
Expression of genes encoding enzymes involved in ABA biosynthesis. Wild and cultivar soybean plants were grown in vitro for 35 days in control condition (22/24 °C), in condition of long middle stress treatment: cold (12/16 °C), heat (34/36 °C), and salinity (90 mM NaCl). In addition, 35-day-old plants of each variant were treated with short hard stresses: cold (4 °C), heat (40 °C), and salinity (150 mM NaCl) for 1 and 4 h. All kinds of stress treatments were repeated three times in three independent experiments (biological replicates). qPCR of each biological replication was performed in three technical replicates. Data are presented as a heatmap calculated from qPCR data as 2^−^^ΔΔCt^. Different colours indicate significantly different means (*p* < 0.05), Fisher’s LSD.

qPCR analysis of *ZE* and *NCED* genes of *G*. *max* was investigated under short- and long-term abiotic stress. Short-term stress exposures were 1 and 4 h at 4 °C, 40 °C, and 150 mM NaCl, and long-term exposure was 35 days at 16 °C, 36 °C, and 90 mM NaCl. For short stress experiments, 35-day-old wild and cultivated soybeans were used; for long-term stress experiments, wild and cultivated soybeans were grown under stress conditions from germination to 35 days. The analysis revealed that *ZE*, a gene encoding zeaxanthinepoxidase, was expressed without any changes in wild and cultivated soybeans under control and stress conditions. *ZE* expression was the lowest among the analysed ABA-related genes (Figure 6, Supplementary Table S5).

Among the three isoforms of *G*. *max NCEDs*, *GmNCED1* is the most related to stress-sensitive *AtNCED3* [29]. qPCR analysis of *GmNCED* expression patterns under stress treatments showed that only the expression of *GmNCED1* was sensitive to stress in all variants (Figure 6, Supplementary Table S5). Interestingly, temperature stress induced *GmNCED1* expression after 4 h, while salinity induced the expression after 1 h. The stress-inducible increase in G*mNCED1* expression in Sfera and wild soybean was significantly higher than that in Hodgson and Hefeng25 (Figure 6, Supplementary Table S5). The expression of two other *GmNCEDs*, *GmNCED2* and *GmNCED5* did not change under stress treatment. However, there was a significant increase in *GmNCED5* expression in Sfera and wild soybean compared with Hodgson and Hefeng25 (Figure 6, Supplementary Table S5).

### 3.6. Effect of ABA Treatment on GmCDPK Expression

ABA is a basic stress-response hormone in plants [30]. A previous study of *GmCDPK* expression after ABA treatment showed that *GmCDPK5* was the most ABA-sensitive isoform [17]. To confirm the high level of stress-induced expression of *GmCDPK5* in the northern wild and cultivated soybeans, we performed the following detailed experiment. Differential expression of *AtCPK1*-related *GmCDPKs* was analysed in wild soybeans with unique primers after treatments with 1 and 5 mM ABA for 1, 4, and 24 h. For this analysis, 35-day-old plants (second trifoliate stage) were grown in vitro to exclude any possible additional effects, such as contamination.

**Figure 7:**
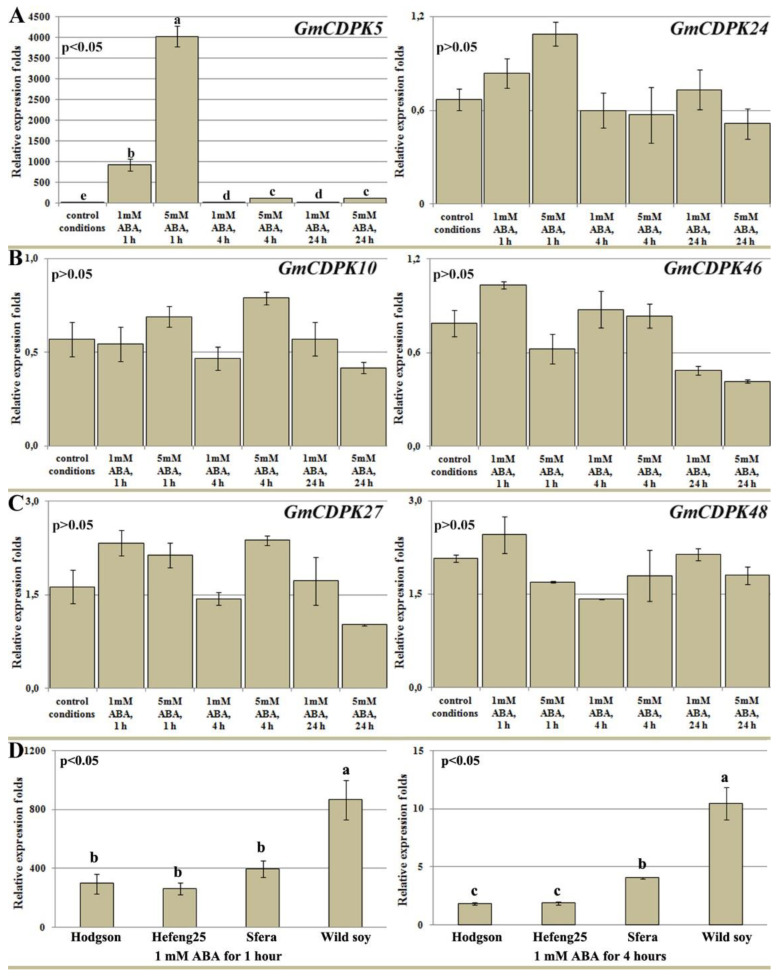
Profiling *GmCDPK* expression after ABA treatment. An ABA treatment test (**A**–**C**) was performed on 35-day-old wild soybeans pants. Plants were sprayed with fresh-prepared 1 mM and 5 mM ABA solutions. mRNA levels of *GmCDPKs* were measured by real-time PCR 1, 4, and 24 h after ABA treatment: (**A**) GmCDPK5/GmCDPK24 paralogs pair; (**B**) GmCDPK10/GmCDPK46 paralogs pair; (**C**) GmCDPK27/GmCDPK48 paralogs pair. *GmCDPK5* expression (**D**) was measured after 1 and 4 h of 1 mM ABA treatment in wild and cultivar soybean varieties (Hodgson, Hefeng25, and Sfera). Plants were grown in vitro for 35 days in control condition (23 °C). ABA treatments were repeated three times in three independent experiments (biological replicates) and qPCR of each biological replicates were performed in three technical replicates. The data are presented as mean ± SE based on qPCR data calculated as 2^−^^ΔΔCt^. Different letters above the bars indicate significantly different of the means in more than two times (*p* < 0.05), Fisher’s LSD.

qPCR analysis showed that among the six *GmCDPKs*, only *GmCDPK5* expression was sensitive to exogenous ABA treatment (Figure 7). Its expression dramatically increased 1000 and 5000 times, compared with control conditions, after 1 h of 1- and 5-mM ABA treatments, respectively. This unexpectedly large difference in expression was due to the low baseline expression of the gene in the control and strong induction by ABA. After 4 h, *GmCDPK5* expression decreased. However, the activating effect of ABA was preserved, it was significant and positively dose-dependent. (Figure 7A). The other analysed *GmCDPKs* were not affected by ABA treatment (Figure 7A–C).

To compare ABA-induced *GmCDPK5* expression in cultivated and wild soybean, the treatment with 1 mM ABA was used for 1 and 4 h (Figure 7D). In cultivated soybeans, the *GmCDPK5* induction after 1 h was half as much as in wild soybeans. After 4 h, *GmCDPK5* expression was twofold higher in Sfera compared with Hodgson and Hefeng25 and fivefold higher in wild soybean, compared with Hodgson and Hefeng25 (Figure 7D).

## 4. Discussion

Calcium-dependent protein kinases (CDPKs) may be considered as promising targets for genetic and bioengineering purposes in plants due to their pivotal role in the process of regulation of plant development and response to stress [6,8,31]. They are involved in stress resistance through direct phosphorylation of ion channels, phosphorylation of different enzymes such as PAL and RBOHD, as well as phosphorylation of hormone signalling components, of which the ABA pathway is the most studied [6,7]. CDPKs assemble signalling proteins in networks defined as signalling complexes or scaffolds [6], which allows the generation of complex responses leading to stress-related polyphenol accumulation, ROS wave propagation, stomatal closure, and other processes allowing plants to adapt biochemical processes to current conditions.

In the present study, expression of the *GmCDPK* genes, which are homologous to the *AtCPK1* gene, a positive regulator in the response to biotic [7,8] and abiotic stresses [9,10,11], was analysed. Phylogenetic and molecular analysis performed in the previous [17,32] and present (Supplementary Figures S2 and S3) studies showed that among 50 isoforms of GmCDPKs, paralog pairs GmCDPK27/GmCDPK48, GmCDPK5/GmCDPK24, and GmCDPK10/GmCDPK46 were the most AtCDPK1-related isoforms. Among them, the GmCDPK27/GmCDPK48 pair had the highest homology, and GmCDPK10/GmCDPK46 pair had a lower homology to AtCDPK1 (Supplementary Figure S3, Supplementary Table S3). The presence of so many close homologues of one isoform is due to polyploidisation and whole-genome duplication events during soybean evolution [33].

Among the studied isoforms, the *GmCDPK5* isoform was the most sensitive to biotic and drought stress treatments, while *GmCDPK27* expression was not significantly changed [17]. In order to identify perspective *GmCDPK* isoforms, the expression patterns of three pairs of AtCDPK1-related paralogs were analysed in wild and cultivated soybeans after abiotic stress treatment. Wild soybeans are more tolerant to adverse climate conditions [34,35,36]; therefore, in breeding, they can be a source of the new elite genes that provide protection against stress [37]. In the present study, a high level of tolerance to extreme high and low temperatures was observed in wild soybeans from the northernmost area (Figure 1 and Figure 2). Only tolerance of Sfera can be compared with wild soybeans. This can be explained by the long period of zoning of parental forms in the conditions of highly adverse climate, since the northern regions of East Asia are one of the oldest places of secondary zoning for soybean cultivars [38]. In contrast, the growth inhibition effect of moderate salinity was similar to cultivars and wild soybeans, although seed germination of wild soybean under high NaCl concentrations was significantly higher, and seeds were viable, in contrast to cultivated varieties. The results demonstrated the high tolerance of whole plants and the persistence of wild soybean seeds from the northern natural habitat under severe and long-term adverse effects.

These findings confirm the importance of using wild soybeans in the breeding process as a pool of genes that can provide high stress resistance, which was lost during domestication. Wild soybeans have considerably higher polymorphisms compared with cultivated soybeans [39,40,41,42,43,44]. It is known that genetic variation in the soybean population is highly associated with ecological adaptability to climate conditions [43]. It has been proposed that wild soybean from the south of the northern regions of East Asia has the highest genetic variation [44] because plants grow under different adverse conditions, including monsoon waterlogging, drought, anomalous low temperatures at the beginning and end of the growing season, and subtropical heating at the middle of the growing season.

The results of a comprehensive analysis of the expression of three pairs of paralogs are consistent with the stress test data. *GmCDPK5* and *GmCDPK10* were the most stress-inducible *GmCDPK* genes in the present study. Moreover, their expression was highly regulated during growth phases. After the trifoliate phase, the expression decreased to an almost undetectable level, while the expression of other studied *GmCDPK* isoforms did not change more than twofold. The existence of these two stress-inducible isoforms at early stages of development might be associated with the need for young plants to be better protected from adverse conditions. The increase in expression of *GmCDPK5* and *GmCDPK10* under severe short-term stress was significantly higher in wild soybeans than in cultivated varieties (Figure 4, Supplementary Table S5). Among cultivated varieties, Sfera had a higher expression level than Hodgson and Hefeng25. Recently, Zhao et al. [33] reported that the expression of surplus paralogs was regulated through trans-regulatory changes. In this case, the level of diversity depends on climatic conditions [44,45]. It can be assumed that adverse climatic conditions in the northern soybean growing area may be the cause of differences in stress tolerance and *CDPK* expression between endemic and ecdemic soybean variants.

ABA is an important stress hormone that regulates the expression of genes involved in acclimation to adverse environmental conditions [18,46]. A growing body of information points to the interaction between CDPKs and ABA regulatory elements in *Arabidopsis* [47]. The current model suggests that altered expression of *CDPKs* affects ABA sensitivity and, consequently, ABA responses [47]. In turn, ABA regulatory elements affect CDPK activity. Much less data are known for soybean plants. In particular, Li et al. [46] reported an increase in the expression of CDPK genes and the level of ABA during drought stress.

Based on the previous study, the most ABA-induced isoform of *GmCDPKs* was *GmCDPK5* [17]. However, there was no comparative analysis of the ABA-induced expression of all six paralogs. Our analysis of the expression of three *CDPK* paralog pairs under ABA treatment at three time points showed detailed dynamics of *GmCDPKs* expression. Among the six studied *GmCDPK* isoforms, only the expression of *GmCDPK5* increased after ABA treatment in a dose- and time-dependent manner. ABA-induced *GmCDPK5* expression was significantly higher in the highly stress-tolerant soybean variety Sphera and wild soybean (Figure 7D). Targeted LC–MS/MS analysis of baseline endogenous ABA at the trifoliate stage showed that wild soybeans and Sfera had nearly twice the ABA of Hodgson and Hefeng25. In the analysis of the expression of genes involved in the basic and stress-inducible ABA biosynthesis, only short-term (1 or 4 h after treatment) cold, heat, and salt treatments induced expression of *GmNCED1* (encoding 9-*cis*-epoxycarotenoid dioxygenase 1, one of the key enzymes involved in stress-induced ABA biosynthesis), which is closely related to stress-dependent *AtNCED3* [20,21]. Expression of other ABA-biosynthesis related genes was not changed. This finding is important addition to a previous investigation, which found that namely *GmNCED1* isoform plays an important role in salt tolerance [48]. The observed increase in *GmNCED1* expression in wild soybean and Sfera was significantly higher compared with Hodgson and Hefeng25. Furthermore, in normal conditions, an increased expression of *GmNCED5* was observed in wild soybean and Sfera compared with Hodgson and Hefeng25.

It was shown recently, that overexpression of *AtNCED3* in *G. max* increased oxidative stress, decreased fermentation in plants exposed to waterlogging, and, ultimately, reduced seed weight and yield [49]. We suggested that, while *GmNCED1* provides an increase in ABA level after stress treatment for stress acclimation, *GmNCED5* probably maintains a basic level of ABA for successful growth and development in normal conditions. Interestingly, the time points for stress-induced activation of *GmNCED1* and *GmCDPK5* expression coincided, especially at 4 h for temperature exposition and 1 h for salinity treatment. These data can indicate a link between ABA-dependent regulation of stress reactions and *GmCDPK5* involvement in ABA-mediated stress responses in soybean plants. The main difference between the *GmCDPK5* and other homologues is its low baseline level of the expression and dramatic rise under stress (including ABA treatment). We suggest that these results point to a role of GmCDPK5-induced target protein phosphorylation in soybean stress tolerance. Given that the Sfera variety has superior yields over non-local cultivars under unfavourable climate conditions [22], it can be assumed that the differences in gene expression described in this work play a role in the adaptive potential of this variety.

## 5. Conclusions

The *GmCDPK5* isoform is the most involved *CDPK* gene in abiotic stress response among three pairs of paralogs that are closely related to AtCDPK1. It may be used for genetic modification or as a selection marker. Wild soybeans from the most northern area may have high genetic potential as a parent material for breeding through hybridisation with cultivated varieties to obtain stress-resistant soybean cultivars.

## Data Availability

The datasets generated during and/or analysed during the current study are available from the corresponding author on reasonable request.

## Supplementary Materials

The following supporting information can be downloaded at: https://www.mdpi.com/article/10.3390/life12101576/s1, Figure S1: Alignment of nucleic acid sequences of *GmCDPKs*, homologues of *AtCDPK1*, Figure S2: Phylogenetic relationship of CDPK proteins from *A*. *thaliana* and the closest AtCPK1 homologues from *G*. *max*, Figure S3: Alignment of amino acid (a.a.) sequences of GmCDPKs (Gm5, 10, 27, 46, and 48), homologues of AtCDPK1, Figure S4: Phylogenetic relationship between CDPK protein of the AtCPK1 (designated as Ak1) and the closest AtCPK1 homologues from *G*. *max* (Gm5, 10, 27, 46, and 48), Table S1: The primer pairs used for the qPCR analysis of the *GmCDPK* genes, Table S2: The primer pairs used for the qPCR analysis of the *G. max* genes of ABA biosynthesis enzymes, Table S3: Percent identity matrix of pairwise sequence identities of *GmCDPKs*, based on nucleic acids (CDS parts) and amino acid multiple sequences alignments computed using Clustal Omega, Table S4: Normalized mean Ct of real time PCR of *GmCDPKs*, Table S5: Normalized expression folds (2^−^^ΔΔCt^) of real time PCR of *GmCDPKs* and ABA biosynthesis genes.
