# Supplementary material for: ABA-Dependent Regulation of Calcium-Dependent Protein Kinase Gene GmCDPK5 in Cultivated and Wild Soybeans
Source: Life (Basel). 2022 Oct 11;12(10):1576. doi: 10.3390/life12101576 (PMC9604703; doi:10.3390/life12101576)
Supplement: Supplementary file 1 [file life-12-01576-s001.zip › life-1931835-supplementary.pdf]

AABA-Dependent Regulation of Calcium-Dependent Protein

Kinase Gene *GmCDPK5* in Cultivated and Wild Soybeans

Galina N. Veremeichik [1], Evgenia V. Brodovskaya [1], Valeria P. Grigorchuk [1], Ekaterina S. Butovets [2], Ludmila M. Lukyanchuk [2] and Victor P. Bulgakov [1,*]

[1] Federal Scientific Center of the East Asia Terrestrial Biodiversity, Far Eastern Branch of the Russian Academy of Sciences, 159 Stoletija Str., 690022 Vladivostok, Russia

[2] Federal Scientific Center of Agrobiotechnology of the Far East Named after A.K. Chaika, 692539 Ussuriysk, Russia

* Correspondence:bulgakov@biosoil.ru; Fax: +7-423-2310193

Supplementary figures and tables

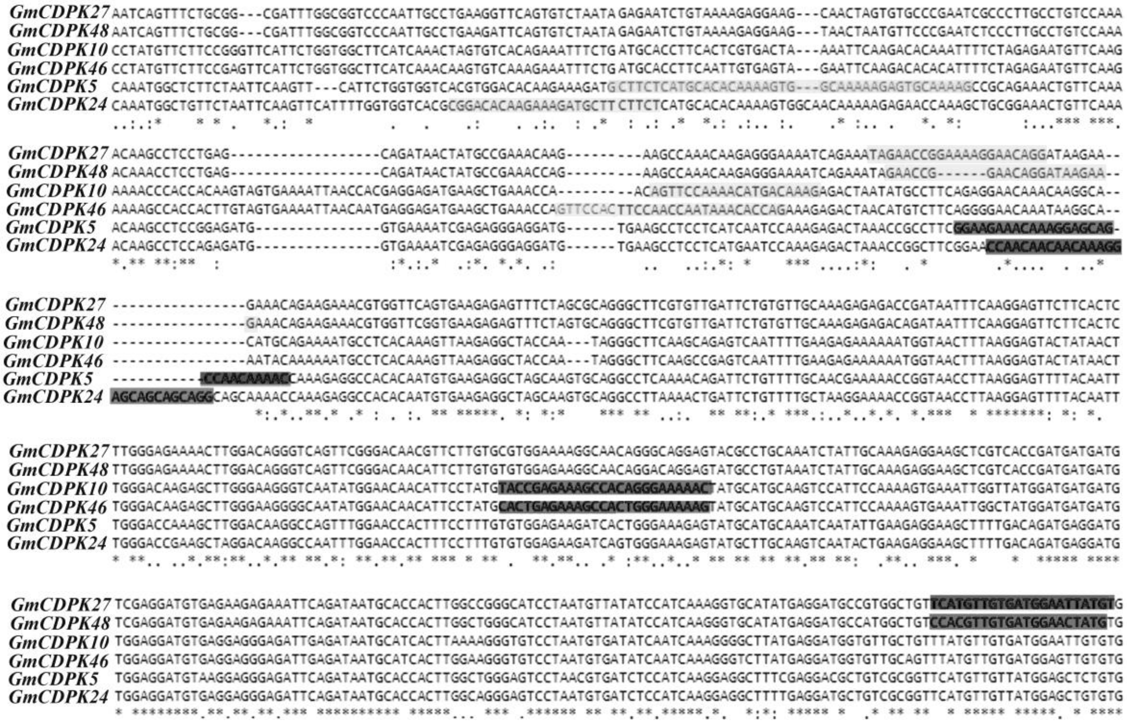

Supplementary Figure S1. Alignment of nucleic acid sequences of *GmCDPKs*, homologues of *AtCPK1*. Forward and reverse primers are highlighted in light gray and dark gray, respectively. The primer pairs were designed at the sites of maximum sequence difference in the pairs of *GmCDPK* paralogs (*GmCDPK5*/*GmCDPK24*, *GmCDPK10*/*GmCDPK46* and *GmCDPK27*/*GmCDPK48*).

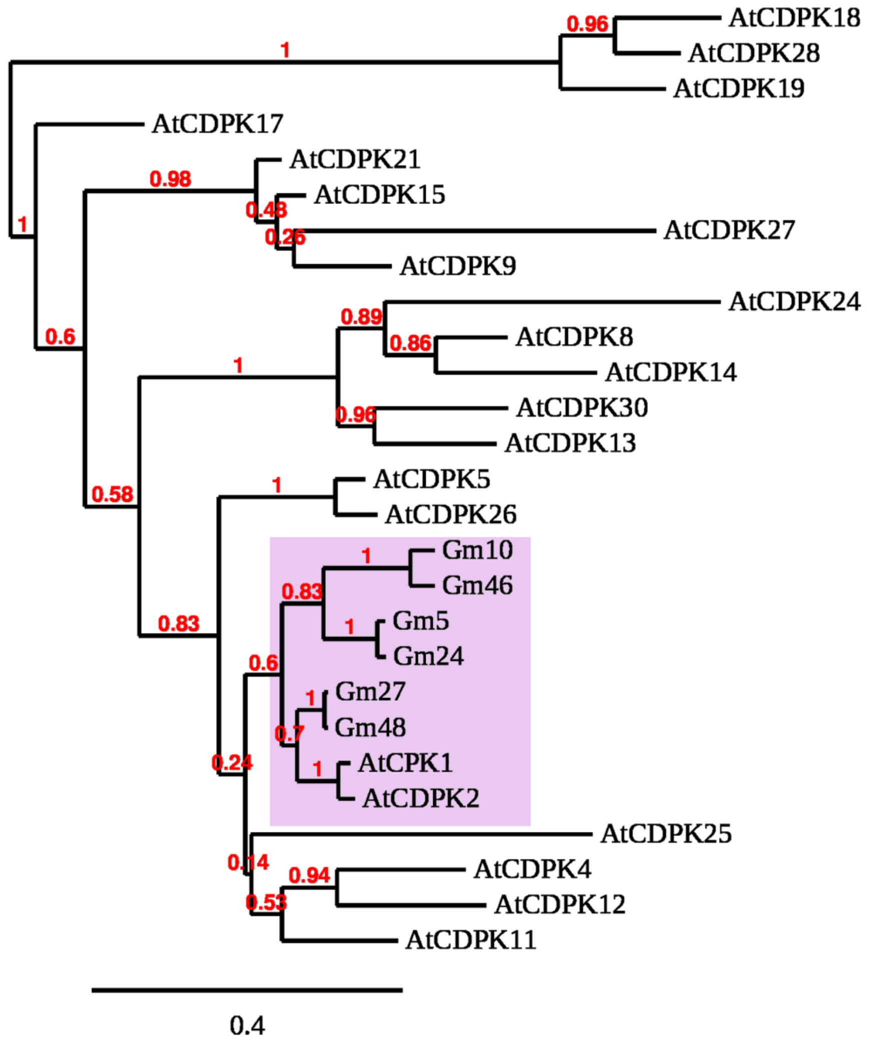

Supplementary Figure S2. Phylogenetic relationship of CDPK proteins from *A. thaliana* and the closest AtCDPK1 homologues from *G. max*.

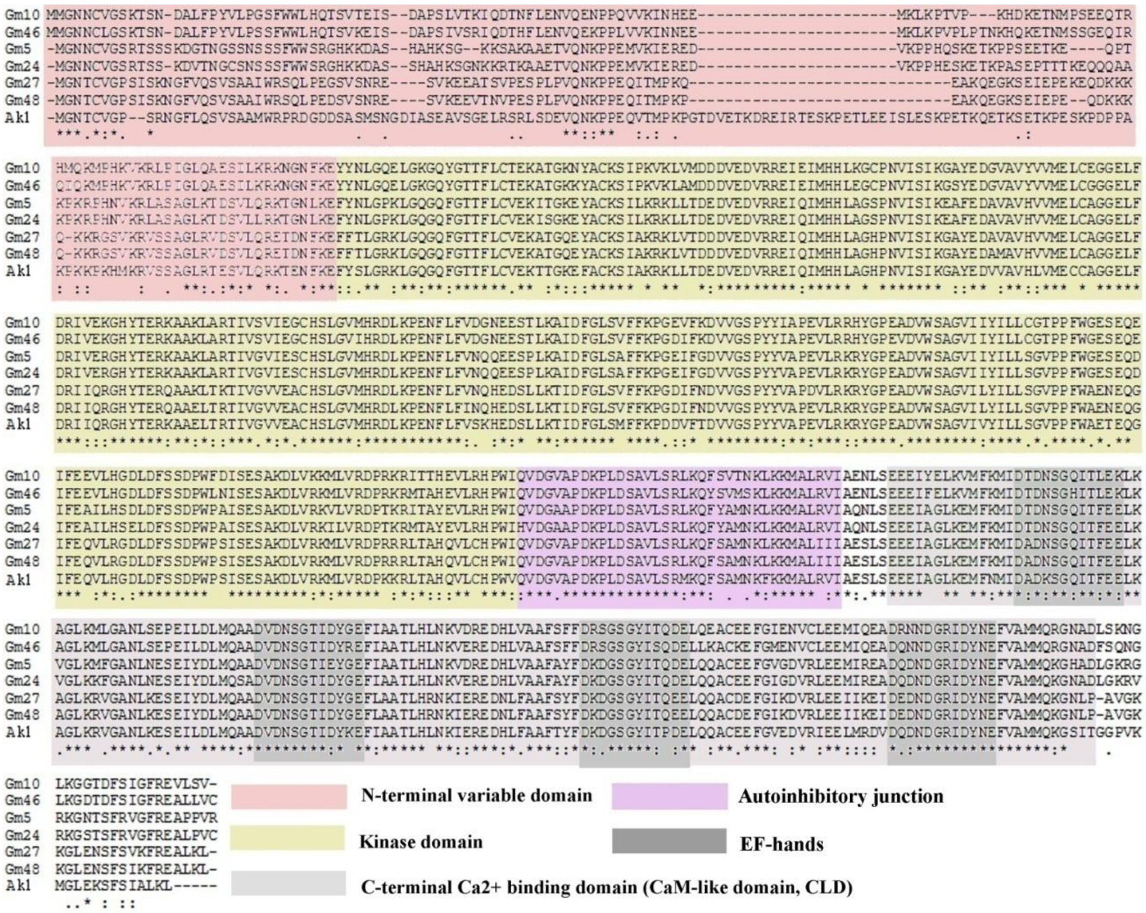

Supplementary Figure S3. Alignment of amino acid (a.a.) sequences of GmCDPKs (Gm5, 10, 27, 46 and 48), homologues of AtCDPK1 (designated here as Ak1). Pink block is a.a. sequences corresponded to N-terminal variable domain; yellow block is a.a. sequences corresponded to Protein kinase domain; purple block is a.a. sequences corresponded to Junction and light gray block is a.a. sequences corresponded to CaM-like domain with EF-hands highlighted in dark gray. All domains were analyzed using Prosite Expasy.

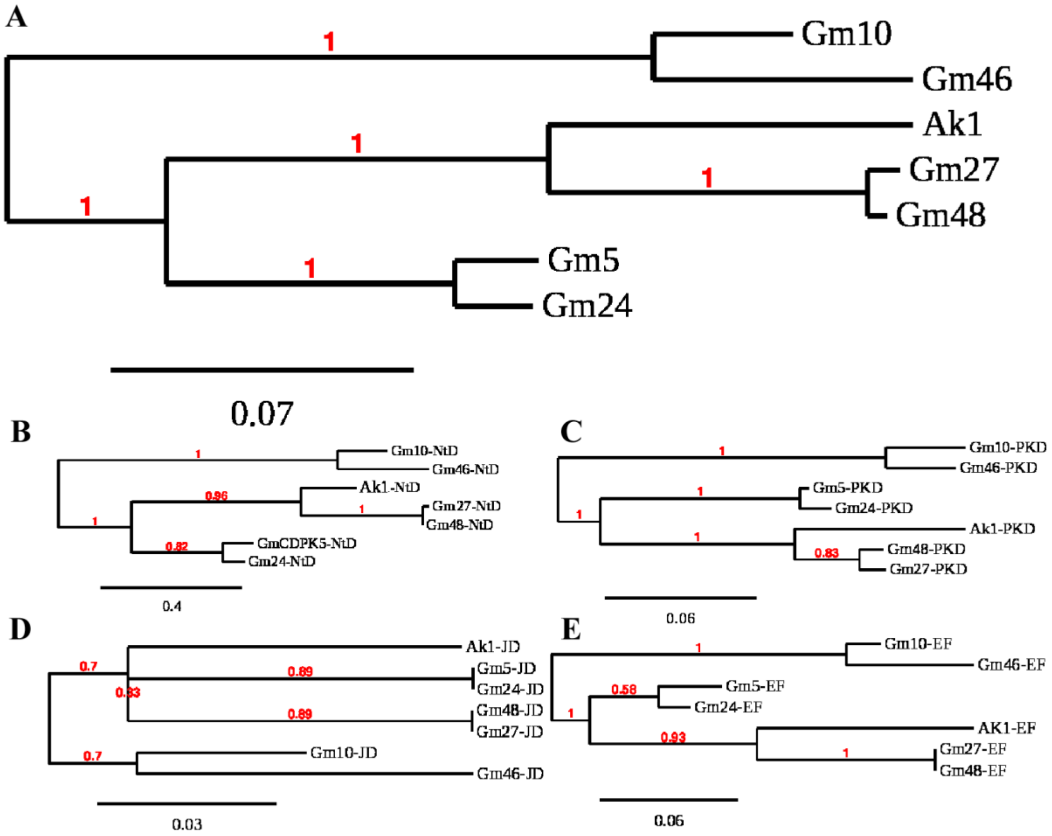

Supplementary Figure S4. Phylogenetic relationship between CDPK protein of the AtCPK1 (designated as Ak1) and the closest AtCPK1 homologues from *G. max* (Gm5, 10, 27, 46 and 48). Full length (**A**) and parts (**B** - **E**) of amino acid sequences of CDPKs were used for phylogenetic analysis. NtD (**B**), parts of a.a. sequences corresponded to N-terminal variable domain; PKD (**C**), parts of a.a. sequences corresponded to Protein kinase domain; JD (**D**), parts of a.a. sequences corresponded to Junction and EF (**E**), parts of a.a. sequences corresponded to CaM-like domain. All domains were analyzed using Prosite Expasy.

Supplementary Table S1. The primer pairs used for the qPCR analysis of *GmCDPKs*

Gene name, Phytozome ID	GenBank accession №	Direct primer (5’-3’)	Reverse primer (5’-3’)	Size, bp
GmCDPK5, Glyma02g34890	XM_003518113	GCTTCTCATGCACACAAAAGTGGCAAAAAG	GTTTTGTTGGCTGCTCCTTTGTTTCTTCC	66
GmCDPK24, Glyma10g10501	XM_028328007	GCGGACACAAGAAAGATGCTTCTTCT	CCTGCTGCTGCTCCTTTGTTGTTGTTGG	92
GmCDPK10, Glyma03g36240.2	XM_003520674	AGTTCCAAAACATGACAAAG	GTTTTTCCCTGTGGCTTTCTCGGTA	26
GmCDPK46, Glyma19g38890.1	XP_003553625.2	GTTCCACTTCCAACCAATAAACACC	CTTTTTCCCAGTGGCTTTCTCAGTG	37
GmCDPK27, Glyma10g23620	XM_003535897	TAGAACCGGAAAAGGAACAGG	CATAATTCCATCACAACATGA	70
GmCDPK48, Glyma20g17020.1	XP_003555695.1	GAACCGGAACAGGATAAGAAG	CATAGTTCCATCACAACGTGG	42

Supplementary Table S2. The primer pairs used for the qPCR analysis of the *G. max* genes encoding ABA biosynthesis enzymes

Gene name	GenBank accession №	Direct primer (5'-3')	Reverse primer (5'-3')	Size, bp
GmZE	ADK26569	GAAGATGATGAAGCACTCGA	CGTAAATCAATCAAGAAGAAG	242
GmNCDE1	XM_014768319	CCCCGCAGACTCCATTTTC	CTCCGATTTCCATTCCTTCTC	382
GmNCDE2	NP_001241251	GACACCACCAGATTCAATATTC	CATCCTCATTCCCATTCCC	329
GmNCDE5	NM_001254687	GAAGAGGCACGAGTACGGAG	CAAATCCATAGCCTCCACAAAC	238

Supplementary Table S3. Percent Identity matrix of pairwise sequence identities the *GmCDPKs* based on nucleic acids (CDS parts) and amino acid multiple sequences alignments computed using Clustal Omega.

	AtCPK1	*GmCDPK5*	*GmCDPK24*	*GmCDPK10*	*GmCDPK46*	*GmCDPK27*	*GmCDPK48*
	Nucleic acid sequences identities, %						
AtCPK1	100	68.45	68.43	66.55	65.98	73.61	73.98
GmCDPK5	68.45	100	94.89	77.03	76.92	69.89	70.05
GmCDPK24	68.43	94.89	100	76.83	76.84	70.05	70.00
GmCDPK10	66.55	77.03	76.83	100	94.36	68.35	68.00
GmCDPK46	65.98	76.92	76.84	94.36	100	67.88	67.65

GmCDPK27	73.61	69.89	70.05	68.35	67.88	100	96.72
GmCDPK48	73.98	70.05	70.00	68.00	67.65	96.72	100
	Amino acid sequences identities, %						
AtCDPK1	100	72.79	71.65	64.89	64.04	80.35	81.50
GmCDPK5	72.79	100	95.22	72.93	71.06	73.43	73.08
GmCDPK24	71.65	95.22	100	72.14	70.97	73.44	73.00
GmCDPK10	64.89	72.93	72.14	100	91.34	66.78	67.01
GmCDPK46	64.04	71.06	70.97	91.34	100	65.74	66.15
GmCDPK27	80.35	73.43	73.44	66.78	65.74	100	98.27
GmCDPK48	81.50	73.08	73.00	67.01	66.15	98.27	100

The closest GmCDPKs to AtCDPK1 are highlighted in green, the most different are in redю The GmCDPKs most distant from each other are highlighted in blue.

Supplementary Table S4. Normalized mean Ct of Real Time PCR of *GmCDPKs*

		Days of cultivation					
		5	15	25	35	45	90
GmCDPK5	Hodgson	31.5	31.1	32.7	34.6	38.9	41.9
	Hefeng25	31.4	30.8	32.3	34.8	38.5	41.8
	Sfera	31.2	30.5	32.5	34.2	38.9	41.7
	Wild	30.7	31.2	32.2	34.3	38.4	41.6
GmCDPK24	Hodgson	31.5	31.4	31.4	31.5	31.8	31.6
	Hefeng25	32.4	31.9	31.5	31.4	31.7	31.4
	Sfera	31.5	31.2	31.8	31.3	31.6	31.2
	Wild	31.6	31.3	31.5	31.8	31.6	31.7
GmCDPK10	Hodgson	31.1	31.5	32.7	34.3	36.9	39.7
	Hefeng25	30.8	31.4	32.3	34.7	36.8	39.3
	Sfera	30.5	31.2	32.5	33.9	36.9	39.2
	Wild	31.2	30.7	32.2	33.9	36.9	39.3
GmCDPK46	Hodgson	30.9	30.4	30.6	30.8	30.7	30.9
	Hefeng25	30.6	30.3	30.7	30.9	30.9	30.7
	Sfera	30.5	30.2	30.8	30.7	30.7	30.7
	Wild	30.8	30.1	30.5	30.5	30.6	30.9
GmCDPK27	Hodgson	26.3	26.5	26.2	26.5	26.6	26.6
	Hefeng25	26.6	26.2	26.5	26.5	26.6	27.5
	Sfera	26.5	26.5	26.8	26.6	26.1	27.1
	Wild	26.2	26.2	26.3	26.6	26.4	27.6
GmCDPK48	Hodgson	27.1	27.2	27.2	27.1	27.1	27.1
	Hefeng25	27.1	27.1	27.1	27.2	27.2	27.3
	Sfera	27.3	27.1	27.5	27.2	27.1	27.3
	Wild	27.2	27.2	27.4	27.1	27.2	27.1

Different colors indicate significantly different means ($p < 0.05$), Fisher's LSD. Data correspond to *GmCDPKs* expression in wild and cultivated (Hodgson, Hefeng25 and Sfera varieties) soybeans plants (**Figure 1**) measured at the different stage of cultivation (5, 15, 25, 35, 45 and 90 days). Dynamic experiments repeated three times.

Supplementary Table S5. Normalized expression folds ($2^{-\Delta\Delta Ct}$) of real time PCR of *GmCDPKs* and ABA biosynthesis genes.

		Normalized expression folds ($2^{-\Delta\Delta Ct}$)									
		Ct 34 – 26		Ct 34 – 27		Ct 27.5 – 26.5		Ct 34 – 25			
		CDPK5	*CDPK24*	*CDPK10*	*CDPK46*	*CDPK27*	*CDPK48*	*ZE*	*NCED1*	*NCED2*	*NCED5*
Hodgson	Control condition	1.2	93	1.2	93	1.2	1.3	1.2	14	4	4.3
	4°C, 1 hour	3	74	1.3	74	1.3	1.4	1.3	14	3.8	4.1
	4°C, 4 hours	6	87	4	87	1.6	1.7	1.06	77	3.2	4.1
	12/16°C, 35 days	1.8	73	1.2	73	1.8	1.3	1.08	13	4.3	4.5
	40°C, 1 hour	7	101	1.3	101	1.7	1.1	1.07	11	4.3	4.2
	40°C, 4 hours	83	111	5	111	1.83	1.1	1.083	31	4.3	4.1
	34/36°C, 35 days	1.5	86	1.2	86	1.5	1.6	1.05	16	4.1	4.1
	150 mM NaCl, 1 hour	11	87	7	87	1.11	1.27	1.1	217	4.5	4.1
	150 mM NaCl, 4 hours	1.7	98	1.2	98	1.7	1.1	1.07	18	4.1	3.9
	90 mM NaCl, 35 days	1.8	95	1.1	95	1.8	0.95	1.08	15	4.5	3.9
Hefeng25	Control condition	1	98	1	98	1.1	0.8	1.1	18	3.9	4.1
	4°C, 1 hour	3.2	88	1.2	88	1.2	1.88	1.2	18	3.9	4.2
	4°C, 4 hours	5	78	5	78	1.5	1.78	1.05	78	3.8	3.9
	12/16°C, 35 days	1.6	95	1.1	95	1.6	1.5	1.06	15	4.5	4.1
	40°C, 1 hour	6.02	108	1.02	108	1.02	1.8	1.02	18	4.3	4.1
	40°C, 4 hours	90	101	6	101	1	1.1	1	81	4.5	4.2
	34/36°C, 35 days	1.8	86	1.2	86	1.18	0.86	1.18	16	4.3	3.9
	150 mM NaCl, 1 hour	12.58	87	8.58	87	1.58	0.87	1.158	227	4.3	4.1

	150 mM NaCl, 4 hours	2.16	98	1.16	98	1.16	0.98	1.16	18	4.8	4.2
	90 mM NaCl, 35 days	1.9	101	1.2	101	1.9	1.01	1.19	11	4.2	4.1
Sfera	Control condition	1.4	112	1.2	112	1.4	1.12	1.14	18	4.1	12.8
	4°C, 1 hour	4.23	98	1.23	98	1.23	0.98	1.123	18	4.2	12.2
	4°C, 4 hours	133.76	87	8.76	87	1.76	0.87	1.176	308	3.9	12.4
	12/16°C, 35 days	2.79	106.9	1.179	106.9	1.79	1.06	1.079	19	3.9	12.4
	40°C, 1 hour	3.2	89	1.2	89	1.2	0.89	1.2	19	3.8	13
	40°C, 4 hours	230	100	10	100	1	1	1.1	77	4.11	12.9
	34/36°C, 35 days	2.28	111.35	1.28	111.35	1.28	1.35	1.28	17	4.1	12
	150 mM NaCl, 1 hour	20	95	10	95	1	0.95	1.20	295	4.3	13.8
	150 mM NaCl, 4 hours	4.66	89.03	1.66	89.03	1.66	1.03	1.166	19.03	4.2	12.9
	90 mM NaCl, 35 days	2.3	98	1.3	98	1.3	1	1.3	18	4.4	14
Wild soybean	Control condition	1	78.773	1	78.773	1	1.773	1	18.773	4.7	17.8
	4°C, 1 hour	6.41	102.28	1.41	102.28	1.41	1.28	1.141	19.28	3.8	18.9
	4°C, 4 hours	320	93	15	93	1	1	1.20	493	4.2	17.9
	12/16°C, 35 days	7.90	99.9	1.190	99.9	0.90	1	1.090	19.9	4.3	18.9
	40°C, 1 hour	5	100	1	100	1	1	1	20	4.3	19
	40°C, 4 hours	210.4	90.06	6.4	90.06	1.4	1.06	1.4	690.06	4.2	22
	34/36°C, 35 days	13.85	100.9	1.185	100.9	1.85	0.9	1.085	20.9	4.1	21
	150 mM NaCl, 1 hour	79.11	110.3	14.11	110.3	1.11	1.3	1.11	410.3	4.2	21
	150 mM NaCl, 4 hours	12	101	1.2	101	1	1	1.2	21	4.2	22
	90 mM NaCl, 35 days	13.28	95.1	1.28	95.1	1.28	1.1	1.28	15.1	3.9	21

These data explain parameters of *GmCDPKs* expression (**Figure 4** in the main text) in wild and cultivated (Hodgson, Hefeng25 and Sfera) soybeans plants measured at 35 day in plants grown *in vitro* in control condition (22/24°C), in condition of long middle stress treatment: cold (12/16°C), heat (34/36°C) and salinity (90 mM NaCl). In addition, 35 days old plants of each variant were treated with short severe stresses: cold (4°C), heat (40°C), and salinity (150 mM NaCl) for 1 and 4 hours.
